# Towards an understanding of the burdens of medication management affecting older people: the MEMORABLE realist synthesis

**DOI:** 10.1186/s12877-020-01568-x

**Published:** 2020-06-05

**Authors:** Ian Maidment, Sally Lawson, Geoff Wong, Andrew Booth, Anne Watson, Hadar Zaman, Judy Mullan, Jane McKeown, Sylvia Bailey

**Affiliations:** 1 School of Life and Health Sciences, Aston University, Birmingham, B4 7ET UK; 2 Nuffield Department of Primary Care Health Sciences, University of Oxford, Oxford, OX2 6GG UK; 3 School of Health and Related Research (ScHARR), University of Sheffield, Regent Court, 30 Regent Street, Sheffield, S1 4DA UK; 4 Medicines Management Department, West Heath Hospital, Birmingham Community Healthcare NHS Trust, Rednal Road, Birmingham, B38 8HR UK; 5 Bradford School of Pharmacy, School of Life Sciences, University of Bradford, N8 Richmond Building, Richmond Road, Bradford, BD7 1DP UK; 6 Australian Health Services Research Institute (AHSRI), iC Enterprise 1, Innovation Campus, University of Wollongong, Wollongong, NSW 2522 Australia; 7 Sheffield Health and Social Care NHS Foundation Trust, Fulwood House, Old Fulwood Road, Sheffield, S10 3TH UK; 8 PPI representative, School of Life and Health Sciences, Aston University, Birmingham, B4 7ET UK

**Keywords:** Medication management, Medication optimisation, Burden, Older people, Multi-morbidity, Polypharmacy, Informal carers, Normalisation process theory, Complexity

## Abstract

**Background:**

More older people are living in the community with multiple diagnoses and medications. Managing multiple medications produces issues of unrivalled complexity for those involved. Despite increasing literature on the subject, gaps remain in understanding how, why and for whom complex medication management works, and therefore how best to improve practice and outcomes. MEMORABLE, MEdication Management in Older people: Realist Approaches Based on Literature and Evaluation, aimed to address these gaps.

**Methods:**

MEMORABLE used realism to understand causal paths within medication management. Informed by RAMESES (Realist And Meta-narrative Evidence Synthesis: and Evolving Standards) guidelines, MEMORABLE involved three overlapping work packages: 1) Realist Review of the literature (24 articles on medication management exploring causality)**;** 2) Realist Evaluation (50 realist-informed interviews with older people, family carers and health and care practitioners, explaining their experiences)**;** and 3) data synthesis and theorising from 1) and 2).

**Results:**

Medication management was viewed from the perspective of ‘implementation’ and structured into five stages: identifying a problem (Stage 1), getting a diagnosis and/or medications (Stage 2), starting, changing or stopping medications (Stage 3), continuing to take medications (Stage 4), and reviewing/reconciling medications (Stage 5).

Three individual stages (1, 3 and 4) are conducted by the older person sometimes with family carer support when they balance routines, coping and risk. Stages 2 and 5 are interpersonal where the older person works with a practitioner-prescriber-reviewer, perhaps with carer involvement.

Applying Normalisation Process Theory, four steps were identified within each stage: 1) sense making: information, clarification; 2) action: shared-decision-making; 3) reflection/monitoring; and 4) enduring relationships, based on collaboration and mutual trust.

In a detailed analysis of Stage 5: Reviewing/reconciling medications, adopting the lens of ‘burden’, MEMORABLE identified five burdens amenable to mitigation: ambiguity, concealment, unfamiliarity, fragmentation and exclusion. Two initial improvement propositions were identified for further research: a risk screening tool and individualised information.

**Conclusions:**

Older people and family carers often find medication management challenging and burdensome particularly for complex regimens. Practitioners need to be aware of this potential challenge, and work with older people and their carers to minimise the burden associated with medication management.

**Trial registration:**

PROSPERO 2016:CRD42016043506.

## Background

### Rationale for the research

The number and proportion of older people in the United Kingdom population continues to increase [1–4], as does multi-morbidity and polypharmacy amongst them [5–7]. This reflects global trends [8]. Multi-morbidity (two or more long term conditions) and polypharmacy (five or more medications or, if less, a complex regime) are inherently complex and challenging to manage [9, 10].

For older people, living with multi-morbidity and polypharmacy can be burdensome [11–15] and may significantly reduce their quality of life [4, 11, 13–18]. Polypharmacy is associated with an increase in drug related adverse events and non-adherence [19–21], both of which can be costly for health services [22–24]. Informal or family carers who support older people find that helping them with complex medication management can be an onerous responsibility [1, 25]. Practitioners, many of whom continue to work with models of illness or in services that are tailored to acute treatment [17, 26], face a growing workload as this population of older people and their family carers require sustained management and support [5, 7, 10, 16, 26]. Practitioners also encounter additional stresses from time and financial pressures, a decline in workforce numbers, as well as the re-organisation of health and care amid changing financial and political circumstances [27–29]. Structural and operational problems persist despite the need for extended, co-ordinated management that supports older people living with multiple long term conditions [26, 30, 31]. Delivery of older people’s health and care appears to be fragmented [4]. NHS England recently launched the National Health Service Long Term Plan to address many of these long standing strategic issues [32, 33].

Current medication management policy, guidelines and interventions in the United Kingdom are predominantly practice and performance-orientated. They are underpinned by two key approaches: adherence, ‘the extent to which the patient’s action matches the agreed recommendations’ [19] and optimisation, ‘a person-centred approach to safe and effective medicines use, to ensure people obtain the best possible outcomes from their medicines’ [34, 35]. In this project, MEMORABLE (MEdication Management in Older people: Realist Approaches Based on Literature and Evaluation), the term ‘medication management’ has been used to encompass the complexity of practices and behaviours in which performance requirements are integrated with older people’s experiences of living with several diagnoses and complex medication regimes. The term acknowledges that medication management is only part of, but contributes to, the full richness and quality of old people’s day-to-day lives.

Despite clarity on many issues surrounding medication management and older people in research, studies frequently adopt a linear, logic model perspective [36], geared to adherence and optimisation for which a behavioural approach is often advocated. These studies have clarified many of the issues surrounding medication management and older people. However, achieving adherence and optimisation continue to be problematic. Many studies adopt a linear, logic model perspective [36] to describe the factors associated with an intervention. However, they do not identify explanatory mechanisms operating between those factors that would better reflect real world, day-to-day complexity, encompassing the many iterative and causal processes geared to the achievement of meaningful outcomes [15, 37]. Such an explanatory approach underpins this research and therefore the potential utility of findings.

### Focus of the research

MEMORABLE aimed to explain medication management from this wider perspective. A realist approach was chosen because it enabled the researchers to work with complexity and understand how medication management works in the real world [38–45]. Systematic reviews examine an ‘averaging’ effect whereas realist reviews explore contexts where particular mechanisms are most likely and least likely to occur. Realism specifically aims to explain how, why and for whom complex interventions such as medication management work, or not i.e. to provide explanations for the causes of phenomena that take into account the influence of context impacting at the individual level. This contrasts with clinical trials that investigate and control selected causal factors to identify effects at a population level.

Medication management was scoped as an extended, complex process rather than a single intervention, using realist methodology to understand how that process might work. Secondary data from a realist review were combined with primary data from a realist evaluation. A theory-informed and causally-structured evidence and experience base was established to better understand and improve medication management, and develop novel multidisciplinary, multi-agency interventions.

## Methods

### Overview

MEMORABLE involved theorising about the intervention by generating and refining programme theory, and testing with data to explain underlying causal processes, the contexts, mechanisms and outcomes within an intervention, to identify what works, for whom, why and in which circumstances [42, 43]. Causal explanations at individual, group and organisational levels account for the influence of contexts on outcomes. By facilitating the understanding of medication management as a complex intervention, realist methodology aligned well with MEMORABLE’s aims and objectives.

**Fig. 1:**
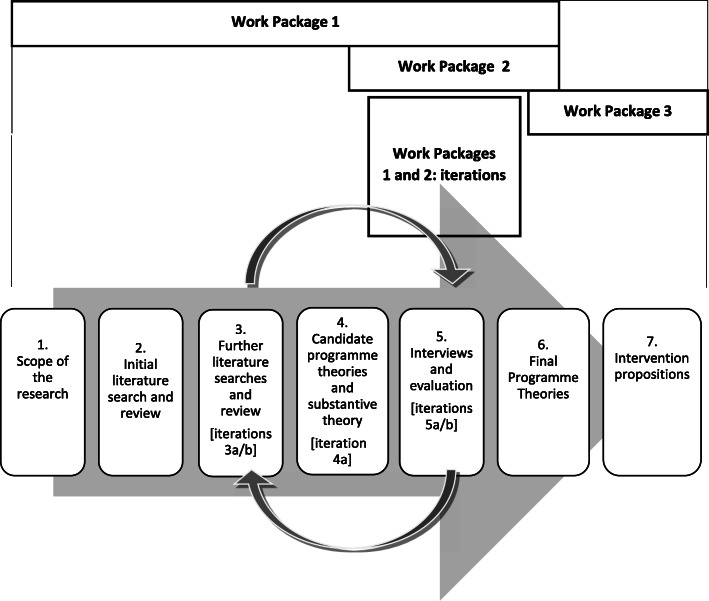
Research Process Summary: Work Packages and Steps

Described in detail in the published protocol [46], MEMORABLE involved three overlapping Work Packages, involving seven, iterative steps: see Fig. 1. The Work Packages and their methods are outlined below in brief:

- **Work Package 1:** Realist Review – Steps 1–4;
- **Work Package 2:** Realist Evaluation – Step 5;

**(Iterations of Work Packages 1 and 2**: Steps 3a and b, 4a and 5a and b); and

- **Work Package 3:** Data synthesis – Steps 6–7.

### Work package 1: realist review

The research began with an exploration of the scope of medication management, by setting out initial programme theories about how it might work. A preliminary systematic literature search was then undertaken: published articles in the English language, 2009–2018, using terms for ‘medication management’, ‘older people’ and ‘long term conditions’ (see Additional File 1). As more than 1000 articles were identified, a further search focussed on the identification of explanatory data in these articles by using specific causal terms: ‘concept’, ‘framework’, ‘model’ and ‘theory’.

Following a realist logic of analysis, patterns of factors identified for their explanatory potential were extracted from the articles and mapped onto a spreadsheet. Qualitative data analysis software (NVivo) facilitated data abstraction and analytical iterations to draft and refine emerging context, mechanism, outcome (CMO) configurations, largely based on interpretations of the data.

As the research progressed, additional searches were carried out on emerging topics of interest, such as burden and shared decision making. There was also a search on substantive theory: Normalisation Process Theory [47]. NPT was specifically chosen for its implementation focus because it articulates the way new practices or activities (for example in relation to medication) are introduced and are made routine or sustained through the work that is done by those involved.

### Work package 2: realist evaluation

50 realist-informed interviews were conducted: older people (n = 13), over the age of 60 years, living in the community with multi-morbidity and polypharmacy and including two older people living with mild dementia amongst other diagnoses; family carers (n = 16) including seven family carers of older people living with dementia and other conditions; and health and care practitioners (*n* = 21). Practitioners included managers and front line staff involved in medication management such as geriatricians, general practitioners, nurses, pharmacists and pharmacy technicians, a social worker, care managers and formal carers. Interviewees were identified through foundation trusts, primary care practices and Join Dementia Research, as well as practitioner and personal contacts.

Participant involvement necessitated approval by the research sponsor, Aston University, a regional Research Ethics Committee by Proportionate Review and the Health Research Authority (approval issued 26th September 2017: REC reference: 17/EE/3057).

Hour long interviews followed a realist-informed schedule to ensure consistency but also the flexibility to explore descriptive and causal accounts as they emerged. Following the same realist logic of analysis, interviewee data describing and explaining how medication management worked for them, day-to-day, were analysed at the level of individual and collective narratives as well as for the underpinning CMO configurations, as in Work Package 1. As the number of interviews analysed this way increased, more robust and detailed patterns of CMO’s emerged directly from these more focused, experiential, causal accounts.

### Work package 3: data synthesis

CMO’s from the literature were synthesised with CMO’s from the interviews, identifying consistent patterns across both data sets as well as other CMO’s of interest because of their explanatory potential. Robust causal accounts elicited from those directly involved in medication management supported revisions to CMO’s generated from the literature where causal links were often lacking. Emerging findings were debated by the Research Team and further iterations of analysis undertaken to revise, refine or reject CMO’s. Generating these increasingly refined causal accounts across both data sets enhanced understanding of the scope of the medication management process as a whole. Also, with the benefit of a number of CMO’s combining both evidence and experiential data, this enabled progressive revision of the initial programme theories from which interventions were proposed. However, because of the quantity and quality of data generated for the whole medication management process, and finite time and funding for the research, the researchers needed to concentrate exploration on areas most likely to yield substantial causal explanations as the basis for improvement proposals. Stage 5: Reviewing/reconciling medication was chosen because it was a well-documented, interpersonal stage, where practitioners had an opportunity to influence what might happen in other stages.

Normalisation Process Theory [47, 48], adapted through the work of Gallagher et al. [49], was applied to the analysis of Stage 5 because it provided concepts that explained the underpinning steps older people and others involved in their care go through to introduce and sustain medication routines, such as sense making and relationships. These steps were mapped across the work on stages, including Stage 5. Thus, this substantive theory augmented the granularity of data analysis, enhancing the drafting and refining of CMO’s.

Final revisions to MEMORABLE’s programme theory were set out in an explanatory framework from which interventions were proposed.

## Results

### Section overview

This section reports the progressive analysis of the literature and interviews, from foundational work on understanding the complexity of medication management to the generation of burden-centred programme theory. It begins with the realist review, augmenting the description of medication management before setting out initial CMO configurations. Findings from the realist evaluation are reported next, highlighting interviewee’s experiences of tasks, routines and outcomes, followed by CMO configurations based on their accounts. Finally, the data synthesis reports the combined analysis of the data sets, addressing the complexity of the medication management process through a five stage, four step, three-loop structure. Informed by Normalisation Process Theory and viewed through the lens of ‘burden’, five burdens were identified from the analysis of CMO configurations for Stage 5: Reviewing/reconciling medication. The progressive focussing of analysis in a realist approach was fundamental to the generation of MEMORABLE’s theoretical framework, concluding this section.

Despite the linear way in which these findings are presented, analysis involved numerous iterations to address the complexity of the topic, refine the scope and processes of medication management and theorise about burden within it.

### Realist review findings: understanding the breadth of medication management (work package 1)

#### Literature on the complexity of medication management

Medication management aligns with the Medical Research Council criteria as a complex intervention [50].

**Fig. 2:**
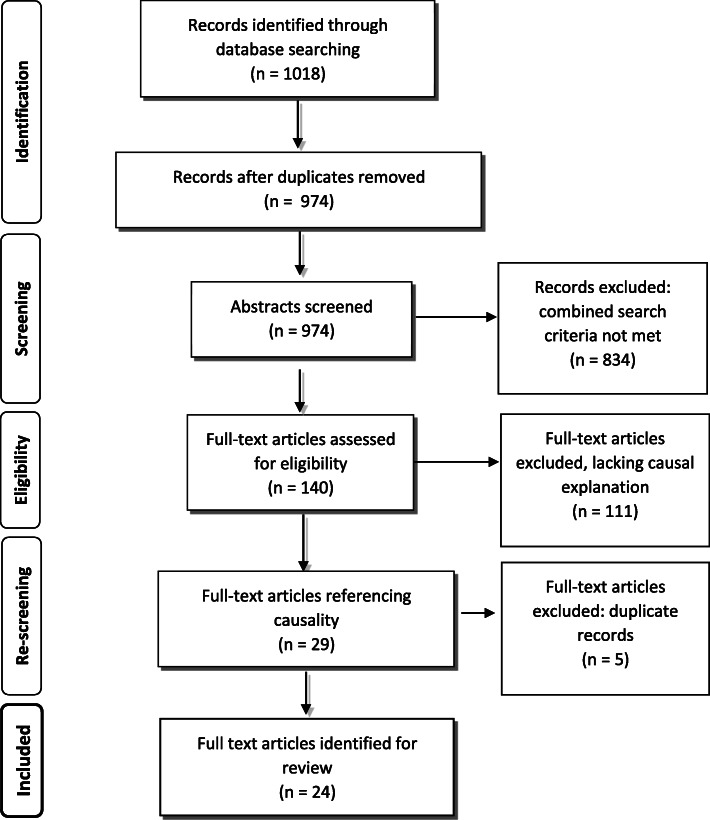
PRISMA Flow Diagram: medication management: developed from Moher et al. [51]

The review screening processes reduced the initial returned articles from 1018 to 24: see Fig. 2 [51]. These 24 articles (from 2009 to 2017), were selected for final analysis being judged as most likely to contain the relevant data needed to build an initial programme theory of medication management. In other words they contained data on: ‘concept’ (*n* = 4) [52–55]; ‘framework’ (*n* = 5) [56–60]; ‘model’ (*n* = 9) [61–69]; or ‘theory’ (*n* = 6) [70–75]. Table 1 sets out details of these documents.

**Table 1:** Medication management articles with the terms concept, framework, model or theory

Authors	Title	Year	Country	Topic	Method
**Concept (n = 4)**					
Cheragi-Sohi, S., Jeffries, M., Stevenson, F. et al. [52]	**The influence of personal communities on the self-management of medication taking: A wider exploration of medication work**	2015	UK	Personal communities involved in medication work	Semi-structured interviews and the construction of Network Diagrams
Fried, T.R., Niehoff, K., Tjia, J. et al. [53]	**A Delphi process to address medication appropriateness for older persons with multiple chronic conditions**	2016	USA	Translating framework concepts into specific strategies to identify and remediate inappropriate regimes, focusing on de-prescribing to reduce medication burden	Modified Delphi process: 3 rounds of anonymised web-based surveys
Naik, A.D., Dyer, C.B., Kunik, M.E. et al. [54]	**Patient Autonomy for the Management of Chronic Conditions: A Two-Component Re-conceptualisation**	2009	USA	Autonomy: decisional (about treatment) and executive (carry treatment plan out) in the context of multiple conditions	Concept development
Upadhayay, J. and Joshi, Y [55].	**Observation of drug utilisation pattern and prevalence of diseases of elderly patients through home medication review**	2011	India	Medication review by pharmacists	Community-based survey
**Framework (n = 5)**					
Bartlett-Ellis, R.J. and Welch, J.L.* [56]	**Medication-behaviours in chronic kidney disease with multiple chronic conditions: a meta-ethnographic synthesis of qualitative studies**	2016	Australia, England, USA	Medication taking and medication adherence behaviours	Qualitative study review
Boskovic, J., Mestrovic, A. Leppee, M. et al. [57]	**Pharmacist competencies and impact of pharmacist intervention on medication adherence: an observational study**	2016	Croatia	Results of pharmacists intervention on adherence in the community	Observational study
Coleman, A [58].	**Medication Adherence of Elderly Citizens in Retirement Homes through a Mobile Phone Adherence Monitoring Framework (Mpamf) for Developing Countries: A Case Study in South Africa**	2014	South Africa	Intervention development: technology	Case Study with qualitative interviews
Schuling, J., Gebben, H., Veehof, L.J.G. and Haaijer-Ruskamp, F.M [59].	**De-prescribing medication in very elderly patients with multi-morbidity: the view of Dutch GP’s. A qualitative study**	2012	Netherlands	Exploring experienced GPs’ views on de-prescribing and involving older people in these decisions	Qualitative study
Yap, A.F., Thirumoorthy, T. and Kwan, Y.H [60].	**Medication adherence in the elderly**	2015	Singapore	Systematic review of the barriers to adherence in the elderly	Case study
**Model (n = 9)**					
Doucette, W.R., Vinel, S. and Pennathur, P.* [61]	**Initial development of the Systems Approach to Home Medication Management (SAHMM) model**	2017	USA	Systems approach to safe and effective home medication management	Model development
Hennessey, B. and Suter, P [62].	**The Community-Based Transitions Model: One Agency’s Experience**	2011	USA	The acquisition and use of health coaching competencies in home care clinicians at health transitions	Model development
Jonikas, M.A. and Mandl, K.D [63].	**Surveillance of medication use: early identification of poor adherence**	2011	USA	Identification of population level adherence and risk of poor adherence	Model development
Khabala, K.B., Edwards, J.K., Baruani, B. et al. [64]	**Medication Adherence Clubs: a potential solution to managing large numbers of stable patients with multiple chronic diseases in informal settlements**	2015	Kenya	Assessment of the people’s care through nurse facilitated Medication Adherence Clubs	Retrospective descriptive study
Kucukarslan, S.N., Lewis, N.J.W., Shimp, L.A. et al.* [65]	**Exploring patient experiences with prescription medicines to identify unmet patient needs: Implications for research and practice**	2012	Canada, USA	Identification and characterisation of patients’ unmet needs when taking prescribed medication	Grounded theory approach to interview content analysis
McHorney, C.A., Zhang, N.J., Stump, T. et al.* [66]	**Structural equation modelling of the proximal distal continuum of adherence drivers**	2012	USA	Identification of adherence drivers	Model development
Lau, Y., Htun, T.P., Chan, K.S. and Klainin-Yobas, P [67].	**Multidimensional factors affecting medication adherence among community-dwelling older adults: a structural-equation modelling approach**	2017	China	Multidimensional factors affecting medication adherence: measuring medication adherence, professional-help relationship and self-care abilities	Exploratory cross-sectional approach using interviews and modelling
Milani, R.V. and Lavie, C.J [68].	**Health Care 2020: Reengineering Health Care Delivery to Combat Chronic Disease**	2015	USA	Modifying the healthcare delivery model to include team-based care in concert with patient-centred technologies	Review
Shepherd, J.G., Locke, E., Zhang, Q. and Maihafer, G [69].	**Health Services Use and Prescription Access Among Uninsured Patients Managing Chronic Diseases**	2014	USA	Identification of relationships between population characteristics, health behaviour and outcomes	Longitudinal quasi-experimental design with convenience sample for assessment and notes review
**Theory (n = 6)**					
Geryk, L.L., Blalock, S.J., DeVellis, R.F. et al. [70]	**Medication Self-management Behaviors among Arthritis Patients: Does Attentional Coping Style Matter?**	2016	USA	Coping styles	Internet based survey
Haslbeck, J.W. and Schaeffer, D [71].	**Routines in medication management: the perspective of people with chronic conditions**	2009	Germany	Routines in medication management along chronic illness trajectory	Semi structured interviews: initial and follow up
Laba, T-L., Lehnbom, E., Brien, J. and Jan, S [72].	**Understanding if, how and why non-adherent decisions are made in an Australian community sample: A key to sustaining medication adherence in chronic disease?**	2015	Australia	Intentional non-adherent decisions and behaviours	Semi-structured interviews and theory-informed iterative thematic framework analysis
Marks, R [73].	**Self-efficacy and arthritis disability: An updated synthesis of the evidence base and its relevance to optimal patient care**	2014	n/a	Self-efficacy, pain and disability, adherence to therapeutic strategies and outcomes, applicable to assessment and treatment	Review and synthesis
Oliveira, C., Helena, J. and Castro-Caldas, A [74].	**Interventions to Improve Medication Adherence in Aged People with Chronic Disease – Systemic Review**	2017	USA, China, Portugal, Italy	Nursing interventions to improve medication adherence in older people with chronic disease	Systematic Review
Skolasky, R.L., Green, A.F., Scharfstein, D. et al. [75]	**Psychometric Properties of the Patient Activation Measure among Multimorbid Older Adults**	2011	USA	Patient Activation Measure (Hibbard): Psychometric properties and model evaluation	Interviews + completion of Measure. Cross-Sectional and latent class analysis

*Articles containing more than one of the search terms

Potentially significant factors but limited causal links were identified in the medication management literature. These factors included the importance of older people’s medication management workload [17, 18, 56], highlighting the influence of diagnoses, symptoms and illness trajectories that overlay ageing processes [11–13, 55]; the medications they take, including high risk drugs, doses and complex regimes [7, 10, 20, 56]; and relationships with prescribers [56, 58, 60]. Without explaining how, these factors were believed to contribute to behavioural responses such as self-efficacy, coping styles and control: ‘personalised, contingent and contextually situated … highly individualised routines and strategies’ [52]. Tentative links between ‘the overburdened patient’, poorer adherence and worse outcomes began to emerge [13–15, 76–81].

The role of doctors [52, 54, 56], and increasingly pharmacists [57, 59, 61] and nurses [52, 56], was central to prescribing [53, 55, 56], de-prescribing [53, 59] and information-giving [56, 59, 62, 65]. Trusted therapeutic relationships [54, 56, 65] were valued for their continuity [82], addressing service and organisational fragmentation [5]. Shared decision making [83–88] was increasingly recognised for the way it enhanced practitioner contacts [9, 34, 89]; this included involving family carers of older people living with dementia [90, 91]. In these circumstances, practitioners appeared more likely to be able to influence older people’s ‘decision architecture’ [92] and therefore what they did at home: ‘enhanc(ing) self-management capacity regarding medication use’ [67].

#### Initial theorising about medication management: setting out preliminary CMO configurations

Semi-structured patterns of causal factors were abstracted from these 24 articles and mapped as preliminary CMO configurations; 10 in total. Examples include the way that polypharmacy increases the risk of adverse drug events through the physiology of ageing (Lit CMO 01); medication adherence is increased when control is given to carers (Lit CMO 02); and adherence to disease specific guidelines increases polypharmacy when practitioners follow evidence-based rather than person-centred practice (Lit CMO 03).

### Realist evaluation findings: increasing the focus on implementation and causality (work package 2)

#### Experiences of medication management: tasks, routines and outcomes

50 interviewees described and then explained their experiences of medication management: see Table 2 for interviewee characteristics.

**Table 2:** Interviewee characteristics

Group	Number	Male/female	Age range / mean	Practitioner roles
**Older people** 60 years+, multi-morbidity, polypharmacy	13	7/6	60–84/ 73	
**Family carers**	16	5/11	N/A	
**Practitioners** (health and care)	21	N/A	N/A	**Doctors:** 2 consultant geriatricians, 2 general practitioners.
				**Nurses:** 5: including a nurse manager, specialist nurses, community matrons.
				**Pharmacy staff**: 3: head of service, clinical pharmacist, pharmacy technician.
				**Social worker:** 1
				**Care providers:** 7: including managers, assessors, team leaders and front-line staff.
				**Strategic manager** (health and care)**:** 1

Analysis validated and extended the results of the review, augmenting the understanding of implementation. Key areas found during analyses were: first, the diverse range of purposeful implementation work, day-to-day tasks and routines interviewees were involved in; and second, outcomes that interviewees identified as important to them.

First, interviewees described their workload, potentially burdensome, including:

- **older people:** making and attending appointments, including organising travel to and from surgeries and hospitals; arranging blood and other tests, and following up results; getting a prescription and having it filled at a local pharmacy; sorting tablets into daily, weekly or monthly containers and typically locating containers in the kitchen to prompt them about tablets that go with food; following a flexible medication management routine to fit with day-to-day life and unexpected events;
- **family carers:** providing physical assistance with appointments, collecting or taking medications; providing cognitive support to ensure medications are taken, ensuring prescriber recommendations followed and sufficient supplies maintained; encouraging and advocating for their family member; and
- **practitioners (role-performance based):** formal carers adhering to local policies and individual care plans for prompting or administering, and reporting medication-taking; a social worker assessing self-medication skills and using Care and Support Planning to meet changing levels of need; general practitioners presenting treatment options to engage older people in decision-making about medication and checking adherence; geriatricians/acute teams getting accurate, timely medication lists on admission and providing revised lists on discharge; nurses and pharmacists carrying out reviews based on a single diagnosis or medication.

Second, interviewees described diverse outcomes associated with medication management that mattered to them and were potentially motivating, including:

- **older person**: *“I can be normal and go out and do things and play with our grandson and cook meals and live a life.”* (OP5);
- **family carer**: *“I want them to keep his condition steady … I can cope with that … and being safe.”* (C14); and
- **general practitioner**: *“The patient still needs and is benefiting from that medication … hopefully doing more good than it is harm … based on current guidance … cost-effective, in terms of a brand or generic prescribing … the patient has the ability to, kind of, understand why they are taking it.”* (P25).

#### Further theorising about medication management: validating and extending CMO configurations

Forty-nine CMOs were developed based on the interviews: 17 CMOs were generated from older people’s interviews, 16 from family carers’ and 16 from practitioners’ accounts. Examples include how older people access healthcare when they think their health or medication is disrupting day-to-day lives and independence, so they regain control (OP CMO 01); family carers increasingly getting involved when they identify health and care problems or gaps, by responding to what is needed (C CMO 3); and how practitioner consistency enables older people to improve the way they manage complexity and risk when dealing with several long term conditions (P CMO 16).

### Data synthesis: moving towards an understanding of burdens in medication management

Further analysis aimed at bringing together both datasets in order to develop a coherent understanding of medication management and culminated in the following key explanatory findings.

#### Medication management: identifying five stages and loops between them

Medication management was refined into five functional stages: (for more information see Additional File 3).

- **Stage 1: Identifying a problem.**
- **Stage 2: Getting a diagnosis and/or medications.**
- **Stage 3: Starting, changing or stopping medications.**
- **Stage 4: Continuing to take medications.**
- **Stage 5: Reviewing/reconciling medications.**

These stages reflect how an older person or their family carer might recognise a health change, perhaps a new symptom (Stage 1) for which they consult a health practitioner (Stage 2). Issued with medication, they then start their new tablets (Stage 3), making them part of their day-to day routine and continuing with their medications (Stage 4), subject to regular review (Stage 5).

Within such an apparently simple scenario, interview data was invaluable in identifying ‘hidden’, dynamic iterations and loops *across* stages (see Additional File 3 for further details).

Interview data also helped to better understand interviewees’ workload and burdens:

- **older people:** the importance of routines and fit with day-to-day life that are indicative of coping with burden, particularly when continuing to take medications in Stage 4; the value attributed to enduring, mutually trusting relationships in Stages 2 and 5; and how practitioner-initiated changes reverberate through existing routines and coping, impacting on initiation work (Stage 3) and sustaining work (Stage 4), with associated emotional, cognitive or behavioural disruptions or loops;
- **family carers:** the stress and risks of their ambiguous ‘informal’ role, evolving and infiltrating all stages, and the lack of training and support they receive. This contrasts with formal carers working under contract, who are trained, supervised and managed to undertake the same, and sometimes more limited, medication management tasks; and
- **practitioners:** the way Stage 5 can loop back to Stage 2 for further diagnostic work or to Stage 3 where medications are changed: See Additional File 3, and the complexity inherent in their formal medication management work to diagnose, prescribe and review in Stages 2 and 5, transacted in time-limited, influential contacts. Health and care practitioners acknowledged increases in caseloads and more complex cases, as well as performance and delivery pressures.

#### Medication management: identifying four steps in each stage

Interview data were also key to exposing another ‘hidden’ aspect of the medication management process; initiating and sustaining work *within* one or more of the five stages, interpreted through and adapted from Normalisation Process Theory [47, 49]:

- **sense making:** finding meaning in events, artefacts or relationships: ‘coherence work’ [47]:
- **older person:***“I don’t think it’s difficult … I quite understand a lot of my drugs as well which helps … interested in the drugs, in what they do and what they’re for … I read the leaflets, yes.”* (OP19);
- **relationships:** interacting with others and valuing continuity: ‘relational work’ [47]:
- **practitioner – general practitioner:***“In terms of decision making, you’re the person best placed to make decisions, if you would recognise things that another clinician might not … what this person’s normally like or how they normally would present … situations where we’ve been here before … if you’ve seen that person a lot you’ll remember that and you’ll remember how you managed it last time. And the medical records don’t give the story.”* (P53).
- **action:** doing tasks: ‘operational work’ [47]:
- **older person, living with mild dementia:***“I know what medication I get, I know that I can get it collected every month, and I’m the one who sticks it in the boxes so I know when to take it … I think the process is important and the routine is important – that’s the key bit really*.” (OP10); and
- **reflection / monitoring**: thinking about what happened and its effect / recording and reporting impact: ‘appraisal work’ [47]:
  - **reflection:**
  - **family carer:***"I’ve walked out of the appointments feeling really sad, thinking “I’m really angry with the way I’ve been treated and the fact that I let it go.”* (C15);
- **monitoring:**
- **practitioner – pharmacist:***“We’d follow NICE guidance with a view to what medication our elderly patients should be on … an area prescribing formulary as well … evidence as to why we have done something.”* (P1).

These steps highlight important processes that underpin behaviours around health and medication, as well as pointing to key individual characteristics and capacities. Thus, in managing their medication and interacting with practitioners, some older people or family carers have capacity to respond and be motivated by information-giving and trust- or confidence-building strategies that impact a sense of control, while others are more action focused. Diminished capacity in any of these steps might lead to being overburdened and not coping with the workload.

#### Theorising on Stage 5 and burden: focused analysis of CMO configurations

***Theorising on Stage 5:*** From the 59 CMO configurations generated by the analysis, four CMOs from the literature directly related to practice-performance in Stage 5: Reviewing / reconciling medications:

- When practitioners carry out a **medication review** using an evidence-based review tool (C), they are more likely to identify and discontinue high risk medications and simplify regimes (O) because they are confident making decisions (M) (Lit CMO 04);
- **Regular medication reviews and transition reconciliations** by experienced practitioners (C), optimises medication management (O), by minimising the risk of treatment related problems (M) (Lit CMO 05);
- When practitioners carry out a **medication review in an older person’s home** (C), they are more likely to identify medication related problems (O), because they understand people’s lived experiences and they take more time (M) (Lit CMO 06); and
- Pharmacists carrying out a **home medication review** (C) are more likely to identify and resolve medication related problems (O), because of their particular expertise and experience (M) (Lit CMO 07).

Additional, more generalisable CMOs were also found to apply to this stage, such as:

- Practitioners, older people, informal carers are likely to make better decisions about medication (O), strengthen their relationships (O) and achieve continuity of care (O), when they collaborate through **shared decision making** (C), because of mutual trust (M) (Lit CMO 08); and
- **Information technology** used by older people, informal carers and practitioners (C) improves access, information sharing and support (O) by reinforcing communication (M) (Lit CMO 09).

***Burden:*** burden was identified as a potential key topic early in MEMORABLE and confirmed as the research progressed, linked to medication management workload and capacity [13, 15, 49, 79]. The researchers established links between concepts of burden, coping and risk, illustrated in Table 3, highlighting a possible burden-coping dynamic and risk association with increasing/high or decreasing/low workload and capacity. The lens of burden was applied to this part of the research.

**Table 3:** Burden, coping and risk in medication management

What capacity does the older person have? *(individual specific)* What is the workload? (*stage specific)*	Increasing / high capacity	Decreasing / low capacity
**Increasing / high workload:** May be high workload per se or may spike at times of change and uncertainty.	**Burden:** coping	**High burden:** not coping – high workload and low capacity risk
**Decreasing / low workload:**	**No burden:** coping	**Burden:** not coping –low capacity risk

Burden was a key concern, such as when older people and family carers described multiple health and care contacts for reviews across different sites, services, teams and practitioners, and the time and effort involved (OP CMO 15). Here the workload involved getting to, and participating in a review at their practice or at a pharmacy; having to visit a phlebotomist for blood tests at their local practice or hospital prior to their review; and seeing a doctor, nurse or pharmacist for the actual review but their doctor or hospital consultant for follow-up. The researchers also identified other hidden burdens, applicable to this stage, where there might be opportunities for mitigation by practitioners. Examples include:

- **knowing what is happening and why:** confidence in services reduces worry when people can rely on them (OP CMO 17);
- **having information:** when informed about complex medications and regimes, people feel in control, making these processes routine and less likely to be forgotten (OP CMO 04);
- **minimising change:** practitioner trust and consistency reduces worry because older people and carers feel understood and supported (OP CMO 12);
- **minimising transitions:** service and practitioner stability helps practitioners and family carers to cope because they are less distracted by fragmentation (P CMO 14); and
- **being engaged:** when family carer’s role, responsibilities and needs are not recognised and systems are unclear, and they are expected to cope, they feel undervalued and unsupported (C CMO 13).

#### Identifying five burdens

In order to consolidate the findings above with a specific focus on the impacts of medication management on older people, further analyses were performed and five burdens were identified from the analysis described above (see Additional File 4 for exemplar quotes illustrating these burdens):

- **ambiguity burden** when the purpose, practice and benefits of reviewing / reconciling medications within medication management are not explained, limiting this stage’s contribution to their health and wellbeing. This aligns with ‘clarification’ work during sense making;
- **concealment burden** due to a lack of information that prevents older people and family carers from understanding, personalising and using what they want or need to know. This relates to ‘information’ work during sense making, contributing to how people use knowledge to increase a sense of personal efficacy, agency, control and coping;
- **unfamiliarity burden** from not seeing the same practitioner consistently. This concerns relationships and establishing foundations of mutual trust through continuity. It also encompasses unfamiliarity with changes to services and staffing in organisations and systems that are in a state of flux, such as from reorganisations and improvement initiatives;
- **fragmentation burden** from being seen by several practitioners working across separate services and organisations, potentially limiting how older people and family carers are understood and how their complex and subtly changing needs are addressed. This relates to a breakdown in face-to-face relationships and communication, including inter-practitioner collaboration, within and between services and organisations, exacerbated by boundaries and transitions; and
- **exclusion burden** when older people and family carers are not recognised for their experience and expertise, nor fully or effectively engaged in decisions that affect their health and care. This concerns action, and the lack of collaboration through shared decision making around common goals.

Burden identification and mitigation were considered to be fundamental aspects of the experience and practice of medication management.

#### MEMORABLE: theoretical framework

**Fig. 3:**
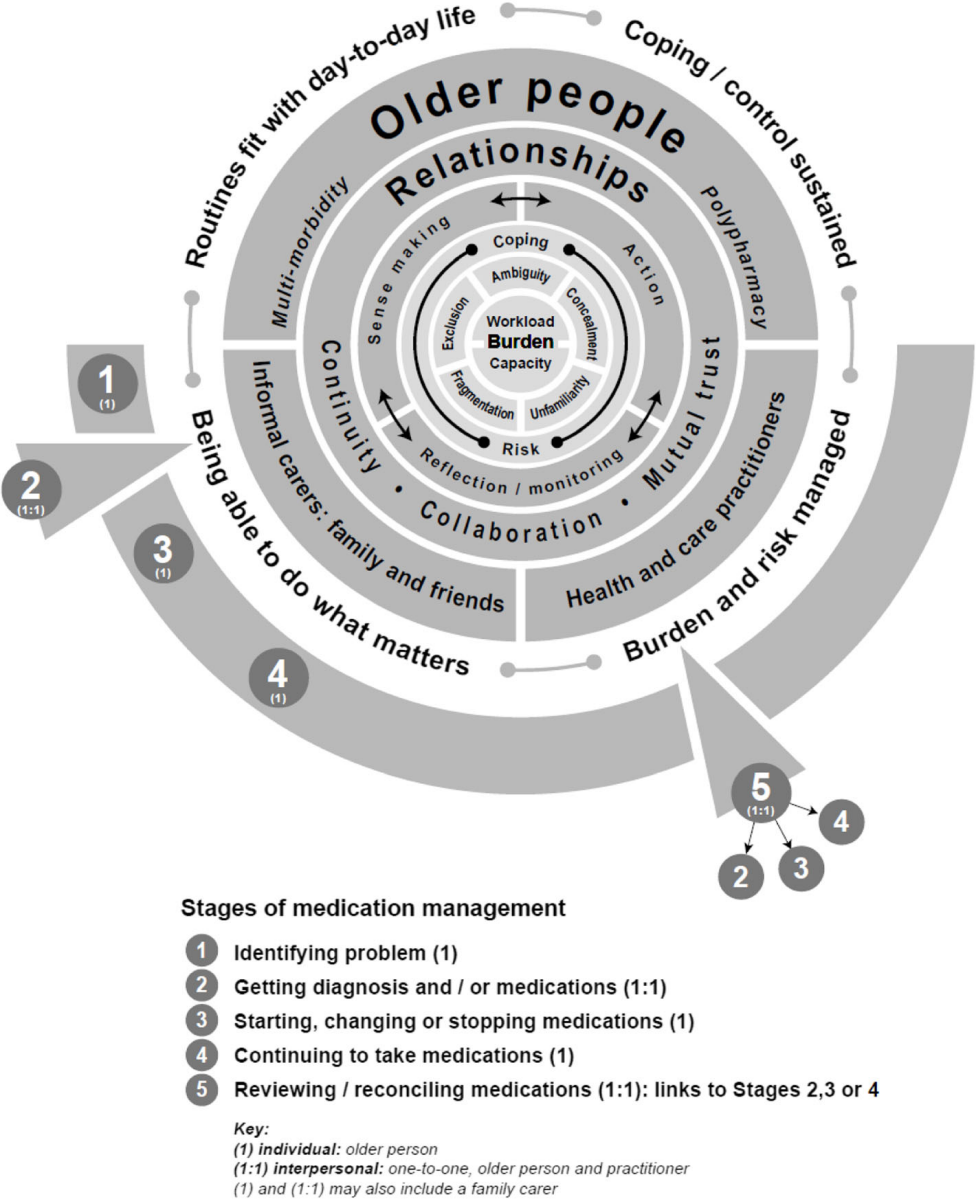
MEMORABLE’s theoretical framework for medication management: understanding burden

The theoretical framework, Fig. 3, is the key output from MEMORABLE. It provides a high level overview and summary of how medication management impacts on those involved that draws together the findings from the above analysis process, highlighting:

- the **focus on participants** in medication management: primarily older people living with multi-morbidity and polypharmacy, but also family carers and practitioners;
- the centrality of **burden (workload and capacity) and types of burden, coping and risk** to medication management; and
- the **implementation steps** that participants are involved in and the importance of the interplay between sense making, action, reflection / monitoring and relationships that are foundational to the work done and outcomes achieved.

The framework also prompts the reader to consider the potential differences in duration of stages, such as Stage 2 typically less than 10 min; Stage 5 perhaps up to an hour; and Stage 4, several months, if diagnoses and medications remain stable.

## Discussion

### Summary of findings

By adopting a realist approach and progressively focusing the synthesis of rich data from evidence and experiential accounts, MEMORABLE has addressed the complexity of the topic, medicine management. The findings have enhanced the understanding of the scope of medication management by establishing a five stage, four step, three loop medication management process. The more detailed analysis of one stage (Stage 5) identified five specific burdens, and generating an evidence and experience-based programme theory set out as a theoretical framework to explain the real world complexity of medication management for and with older people.

### Implications of the research findings

MEMORABLE was conceived as the first study of medication management in older people to use realist methodology. It established that medication management is neither ‘one thing’ nor simply cause and effect. It is a complex implementation process, operating across stages, steps and loops, and at multiple levels, in which older people, family carers and health and care practitioners engage, in various ways and at various times. MEMORABLE acknowledges an apparent tension between different participants’ goals and outcomes.

MEMORABLE has highlighted the importance of individual experience and interpersonal work that directly addresses burden mitigation, through workload, capacity, coping and risk, involving information, clarification and support, transacted through effective shared decision making, and based on relationship continuity and mutual trust.

MEMORABLE reaffirms the place of older people and their experience at the centre of medication management, acknowledging their individuality, ingenuity, sense of purpose and routine-reliance in an attempt to cope with the burden of living with multi-morbidity and polypharmacy.

One of the key, perhaps, the key finding from MEMORABLE was that managing medication, particularly complex regimens, can be very burdensome, impacting on the day-to-day lives of older people and family carers. These burdens are often hidden from practitioners. Practitioners need to make a conscious effort to be aware of the burdens associated with medication management and should work with older people and their carers to reduce them. As a practical example, when a new treatment is considered (Stage 2) practitioners should consider the burden of managing the medication (Stages 1, 3 and 4) just as they consider the side effect potential.

MEMORABLE has highlighted two key practice gaps and generated evidence for addressing burdens: providing information to reflect the individual experience of managing multiple diagnoses and medications in this population; and, identifying those at greatest risk of not coping with the burden of medication management who would benefit from timely referral for additional help and support. Both have the potential to improve medication management, outcomes and satisfaction, benefitting those involved and health and care services.

### Strengths and limitations of the study

#### Strengths

Benefitting from realist expertise amongst the researchers, MEMORABLE has addressed the complexity of medication management using a credible and appropriate methodology. It has demonstrated the advantages of combining data from a realist review with a realist evaluation to significantly strengthen reasoning about causality. Indeed the key strength and novelty of MEMORABLE is the thorough inclusion of the views of older people and their family carers on the challenges associated with medication management.

This approach proved invaluable in overcoming the limited data on causation from existing documents on this topic.

#### Limitations

Grappling with the complexity of the topic was time consuming. The researchers were generating a causal understanding of the topic as the interviews began. It was not possible to refine the interview schedule to focus on the evolving programme theory, and more specifically, Stage 5 and burden.

Also, detailed analysis was confined to a single stage of the medication management process, in line with prioritisation of programme theory which characterises realist approaches, rather than across all five stages.

### Future research directions

Two possible avenues of the development of future interventions have been identified by the researchers, noted in Implications, above. The researchers are planning to take these forward, using collaborative, research-into-practice approaches. However, they recognise the challenges of introducing and embedding more individually responsive, evidence and experience-based approaches to medication management in services, organisations and systems that are stridently performance-driven and change-fatigued.

MEMORABLE has highlighted the plight of informal or family carers, clearly identifying the burden associated with the lack of training and support they receive in comparison with formal carers to carry out the same medication management tasks. Further work in this area is urgently needed.

Additionally, COVID-19 may increase the burden associated with the practical aspects of medication management and make it more challenging for older people and their family carers to obtain appropriate support. Further research on the impact of COVID-19 is required.

Acknowledging the inherent complexity, future research on burdens in medication management should also further investigate how different contextual factors in different patient, family carer and practitioner groups impact day-to-day medication management.

Finally, having established a programme theory for Stage 5, the researchers are seeking to transfer this theory to all stages of medication management. A research proposal for this important realist inquiry is being developed as a platform for further translational projects.

### Comparison with existing literature

The researchers identified only a small number of articles on this topic, none using an explanatory, realist approach. Detailed data on outcomes was lacking and few papers explained the contributions of family carers or social care practitioners to medication management. MEMORABLE offers a potentially significant explanatory realist approach to start to address these gaps. MEMORABLE seeks to establish a nuanced understanding of the concept of burden to add to the extensive literature on the topic. It also adds an applied, exemplar study to the substantial body of work on Normalisation Process Theory.

## Conclusions

Medication management embodies complexity, characterised by open, dynamic systems and processes, engaging diverse individuals, sometimes working alone and occasionally together towards potentially conflicting outcomes. Older people and family carers often find the complexity and burdens associated with medication challenging to manage on a day-to-day basis. Practitioners need to be aware of this potential challenge associated with medication management and its impact on daily lives, and work with older people and family carers to reduce the burdens associated with medication management.

Medication management research can understand this complexity by combining explanatory accounts from multiple perspectives with causally structured evidence in ways that are theory-informed and theory-generating. Centred on the day-to-day experiences and challenges facing all those involved, such a real world approach is vital to developing ways to improve this critical aspect of health and care.

## Supplementary information

**Additional file 1.** MEMORABLE search concepts

**Additional file 2.** MEMORABLE: Interview Schedules: key content of the interview schedules

**Additional file 3.** Five stages of medication management with loops

**Additional file 4.** For exemplar quotes illustrating the five burdens

## Data Availability

This is a qualitative study and therefore the data generated is not suitable for sharing beyond that contained within the report. Further information can be obtained from the corresponding author.

## Abbreviations

CMO configurations: Context, mechanism, outcome configurations: sets out the relationship between causal components that explain what causes an outcome to happen and in which context: Lit CMO – from the literature; OP CMO – from older people; C CMO – from family carers; P CMO – from practitioners
MEMORABLE: Medication Management in Older people: Realist Approaches Based on Literature and Evaluation
Medication management: What older people do to deal with medication and make it part of their day-to-day lives, with or without the support of informal carers. Also, what practitioners do to support older people with the treatment of their co-morbidities and to maintain their health.
NPT: Normalisation Process Theory
Programme theory: A programme theory refers to an abstracted description and/or diagram that lays out what a program (or family of programs or intervention) comprises and how it is expected to work.
Stage 1 (medication management process): Identifying problem
Stage 2 (medication management process): Getting diagnosis and/or medications
Stage 3 (medication management process): Starting, changing or stopping medications
Stage 4 (medication management process): Continuing to take medications
Stage 5 (medication management process): Reviewing /reconciling medications
