# Supplementary material for: Towards an understanding of the burdens of medication management affecting older people: the MEMORABLE realist synthesis
Source: BMC Geriatr. 2020 Jun 5;20:183. doi: 10.1186/s12877-020-01568-x (PMC7272211; doi:10.1186/s12877-020-01568-x)
Supplement: Supplementary file 1 — Additional file 1. MEMORABLE search concepts [file 12877_2020_1568_MOESM1_ESM.docx]

**Additional file 1: MEMORABLE search concepts**

| **Topic** | **Search terms** |
| --- | --- |
| Medication management | Medication adherence – polypharmacy – medication management – medicines optimisation – concordance – compliance – adherence - regimen  Health services misuse / inappropriate prescribing – drug prescriptions / practice patterns – physicians / inappropriate prescribing – drug utilisation / medication errors – de-prescribing |
| Older people | Age/aged |
| Long term conditions | Chronic disease |
