# Supplementary material for: Towards an understanding of the burdens of medication management affecting older people: the MEMORABLE realist synthesis
Source: BMC Geriatr. 2020 Jun 5;20:183. doi: 10.1186/s12877-020-01568-x (PMC7272211; doi:10.1186/s12877-020-01568-x)
Supplement: Supplementary file 2 — Additional file 2. MEMORABLE: Interview Schedules: key content of the interview schedules [file 12877_2020_1568_MOESM2_ESM.docx]

**Additional file 2: MEMORABLE: Interview Schedules: key content of the interview schedules**

**Participant identifiers:** confirming participant and interview details, including unique code used to anonymise the participant.

1. **Baseline demographic data:** *(for monitoring purposes)*: including ethnicity.
2. **Pen portrait:** home circumstances, community circumstances, access to and use of technology, functional status relevant to medication management – sight, hearing, swallow, dexterity, mobility (indoor/outdoor), health and medication.
3. **Finding out about the general experience of medication management:**

**Process of managing medication:**

- *(exploring the intervention*): understanding of medication management (*reference to five stages*), key people involved and doing what; and
- *(exploring what works, for whom, why and in which circumstances*): what currently makes medication management easier / more difficult (*with how and why for explanatory detail*), what would make it better or easier in the future (*solution focus, with how and why for explanatory detail)*

**Outcomes from taking medication:**

- *(generating explanatory accounts, working back from outcomes):* what are the outcomes that matter to that individual, are they achieved, who or what makes it easier / more difficult to achieve those outcomes *(doing what, how and why for explanatory detail, anything else),* who or what would make it better or easier in the future to achieve those outcomes (*solution focus, with how and why for explanatory detail, anything else)*

1. **Eliciting responses to explaining experiences of medication management:** *(responses to candidate programme theories)*: ‘You have told us about your experiences of…’ (*summarise to bridge to programme theory) >* ‘In the research team we have been thinking about…’ *(describe programme theory in detail appropriate to that individual and their experiences) >* ‘What do you think about this *(summary title of programme theory)*? When does it affect you most (*enable discussion of* *detail:* *what, who, why and in which circumstances).*
2. **Anything else arising from the interview.**

NB The schedules for informal carers and practitioners shared this common content but were flexed:

- informal carers provided data on themselves as well as the person they cared for; and
- practitioners scoped their role, expertise and experience, and what informed their practice and provided data on themselves and the older people and/or informal carers they work with.
