# Supplementary material for: Towards an understanding of the burdens of medication management affecting older people: the MEMORABLE realist synthesis
Source: BMC Geriatr. 2020 Jun 5;20:183. doi: 10.1186/s12877-020-01568-x (PMC7272211; doi:10.1186/s12877-020-01568-x)
Supplement: Supplementary file 3 — Additional file 3. Five stages of medication management with loops [file 12877_2020_1568_MOESM3_ESM.docx]

**Additional file 3: Five stages of medication management with loops**

| **Stage** | **Stage 1**  Identifying problem | **Stage 2**  Getting diagnosis and/or medications | **Stage 3**  Starting, changing or stopping medications | **Stage 4**  Continuing to take medications | **Stage 5**  Reviewing / reconciling medications |
| --- | --- | --- | --- | --- | --- |
| **Who / Doing what** | **Older person** **(1)** identifies something is wrong | **Older person and practitioner** **(1:1)** agree what is wrong, how to treat it. A prescription is issued and filled | **Older person** **(1)** adjusts daily medication routine to include new medication or adjust or omits current medication  *^disruption loops^* ^loopldisruption loopon loops^ | **Older person** **(1)** fits new routine into day-to-day life  *^diagnosis loop^*  *^medication loops^* | **Practitioner** confirms medication safety and efficacy  **Older person and practitioner** **(1:1)** agree appropriateness, adherence and fit with day-to-day life |
|  | **Family carers can be involved in any or all stages** | | | | |

**For further explanation on the stages:**

**Stage 1: Identifying a problem:** deciding that something is wrong and acting on it, such as arranging to discuss it with a pharmacist, nurse or doctor.

**Stage 2: Getting a diagnosis and/or medications**: sharing knowledge and decisions, agreeing what is wrong and how to treat it, issuing and filling a prescription if necessary.

**Stage 3: Starting, changing or stopping medications:** a single action to either start a new medication, or adjust or stop existing medications.

**Stage 4: Continuing to take medications:** making new medication part of an established routine in an older person’s day-to-day life and following that routine consistently.

**Stage 5: Reviewing/reconciling medications:** sharing knowledge and decisions, agreeing how medications and medication management are working, and as a result:

- - continuing medications as before: reverting to Stage 4 (medication loop);
  - starting or adjusting medications, including stopping: reverting to Stage 3 (medication loop); or
  - getting a new diagnosis: reverting to Stage 2 (diagnosis loop).

*Family carer involvement may be introduced and gradually increase across all stages Formal carer support may be introduced in stages where need has been established.

The models also incorporates loops:

**Medication loops:** such as from Stage 5 to Stage 4 where a review recommends continuing medications as before or from Stage 5 to Stage 3 where the advice is to adjust or stop existing medications or start new ones.

**Diagnosis loops:** or from Stage 5 to Stage 2 where a new diagnosis is required.

Older people may also experience multiple ‘**disruption loops’** between Stages 3, 4 and 1, associated with changes or challenges to what they are currently doing. These are key decision and action points for the older person and their family carers, resolved by drawing on their knowledge, confidence and preferences about their own complex and unique combination of diagnoses and medications.
