# Supplementary material for: Towards an understanding of the burdens of medication management affecting older people: the MEMORABLE realist synthesis
Source: BMC Geriatr. 2020 Jun 5;20:183. doi: 10.1186/s12877-020-01568-x (PMC7272211; doi:10.1186/s12877-020-01568-x)
Supplement: Supplementary file 4 — Additional file 4. For exemplar quotes illustrating the five burdens [file 12877_2020_1568_MOESM4_ESM.docx]

**Additional file 4: for exemplar quotes illustrating the five burdens**

**Example of ambiguity burden:** “It’s usually 6 months now I think. Something like that….my next one is due in April. So I won’t be able to get these (medications) again until I’ve been reviewed in April… When they say review, sometimes it’s the pharmacist you see…. not necessarily a doctor. In fact, well there’s a practice nurse and a practitioner or there’s a pharmacist or a GP and you never know which one you’re going to get… I mean if you’re taking things, you ought to be reviewed. But as far as I’m concerned once a year is enough.” OP29 – older person.

**Example of concealment burden:** “So many people, I don’t think they necessarily want anything… I think they’re just so used to be told this is what they need to take… People want to have a bit more control over things sometimes, you know what I mean. It feels that they are back in control of it and able to manage it.” P18 – clinical pharmacist

**Example of unfamiliarity burden:** “They didn’t know him (my father)… because they have all just recently changed which is a bit unfortunate… And he said ‘They don’t know me. They don’t listen to me. They don’t ask me how I am. So I am not going to sit there and tell them how I am feeling when they know nothing about me’.” C12 – informal carer.

**Example of fragmentation burden:** “The thing is it’s knowing what facilities and who’s available out there, I mean coming into the community I’m finding people that I didn’t even know, and their roles, existed… But then nobody talks and checks upon each other unless you go to sort of a national conference or somebody just happens to find out.” P33 – practitioner, senior community nurse

**Example of exclusion burden:** “You have to listen to the people that are there all the time doing it.” C11 – informal carer.
